# An oncogenic enhancer promotes melanoma progression via regulating *ETV4* expression

**DOI:** 10.1186/s12967-024-05356-8

**Published:** 2024-06-07

**Authors:** Junyou Zhang, Qilin Wang, Sihan Qi, Yingying Duan, Zhaoshuo Liu, Jiaxin Liu, Ziyi Zhang, Chunyan Li

**Affiliations:** 1 School of Engineering Medicine, Beihang University, Beijing, 100191 China; 2 Key Laboratory of Big Data-Based Precision Medicine (Ministry of Industry and Information Technology), Beihang University, Beijing, 100191 China; 3 School of Biological Science and Medical Engineering, Beihang University, Beijing, 100191 China; 4 Beijing Advanced Innovation Center for Big Data-Based Precision Medicine, Beihang University, Beijing, 100191 China

**Keywords:** Enhancer, *ETV4*, Transcriptional regulation, Tumor progression, Melanoma

## Abstract

**Background:**

Enhancers are important gene regulatory elements that promote the expression of critical genes in development and disease. Aberrant enhancer can modulate cancer risk and activate oncogenes that lead to the occurrence of various cancers. However, the underlying mechanism of most enhancers in cancer remains unclear. Here, we aim to explore the function and mechanism of a crucial enhancer in melanoma.

**Methods:**

Multi-omics data were applied to identify an enhancer (enh17) involved in melanoma progression. To evaluate the function of enh17, CRISPR/Cas9 technology were applied to knockout enh17 in melanoma cell line A375. RNA-seq, ChIP-seq and Hi-C data analysis integrated with luciferase reporter assay were performed to identify the potential target gene of enh17. Functional experiments were conducted to further validate the function of the target gene *ETV4*. Multi-omics data integrated with CUT&Tag sequencing were performed to validate the binding profile of the inferred transcription factor STAT3.

**Results:**

An enhancer, named enh17 here, was found to be aberrantly activated and involved in melanoma progression. CRISPR/Cas9-mediated deletion of enh17 inhibited cell proliferation, migration, and tumor growth of melanoma both in vitro and in vivo. Mechanistically, we identified *ETV4* as a target gene regulated by enh17, and functional experiments further support *ETV4* as a target gene that is involved in cancer-associated phenotypes. In addition, STAT3 acts as a transcription factor binding with enh17 to regulate the transcription of *ETV4*.

**Conclusions:**

Our findings revealed that enh17 plays an oncogenic role and promotes tumor progression in melanoma, and its transcriptional regulatory mechanisms were fully elucidated, which may open a promising window for melanoma prevention and treatment.

**Supplementary Information:**

The online version contains supplementary material available at 10.1186/s12967-024-05356-8.

## Background

Melanoma, caused by the overproliferation of abnormal melanocytes, is one of the most aggressive and treatment-resistant form of skin cancer [1]. Although targeted therapy and immune checkpoint inhibitor therapy have prolonged the survival of patients with advanced melanoma, the 5-year overall survival rate for advanced melanoma is only about 20% [2]. Therefore, early diagnosis and targeting on the key drivers of melanoma initiation and progression are demanding.

Transcriptional regulation, as a key process in gene expression, involves the binding of transcription factors to *cis*-regulatory sequences, such as enhancers, to spatiotemporally controlling gene expression [3–6]. Enhancer dysfunction due to point mutations or structural variants causes aberrant gene expression in cancer [7, 8]. Moreover, genome-wide association study (GWAS) reveals that a large fraction of cancer-associated risk single nucleotide polymorphisms (SNPs) fall within the enhancer region [5, 9–13]. For example, Gao et al. identified a susceptibility SNP rs11672691 in prostate cancer, which is located in an enhancer element and alters the binding site of the transcription factor HOXA2. Due to HOXA2 binding to risk allele G of rs11672691, the transcriptional levels of *CEACAM21* and *PCAT19* are elevated, which consequently promote prostate cancer progression [13].

Recent advances in high-throughput sequencing and its application in enhancer research have deepened our understanding in enhancer and its central role in development and human diseases. Based on enhancer RNA (eRNA) expression and specific histone modifications, such as histone H3 lysine 27 acetylation (H3K27ac) and H3 lysine 4 mono-methylation (H3K4me1), active enhancers can be characterized across the human genome. Large genomic projects, such as ENCODE, FANTOM, Roadmap Epigenomics, provide valuable data resources for enhancer-related research [5, 14–18]. Furthermore, owing to rapid development in gene editing technologies, particularly CRISPR/Cas9 technology, efficient genomic and epigenomic editing of enhancers is practical, enabling the analysis of functional enhancers [19–23]. Although thousands of active enhancers have been identified and validated in the human genome, little is known about their regulatory mechanisms and functions.

ETV4 is a member of the E26 transformation-specific (ETS) transcription factor family [24, 25]. Previous studies have been reported that the ETS transcription factor family plays a critical role in the regulation of tumor initiation and progression, including proliferation, apoptosis, and metastasis [26–28]. *ETV4* is well characterized as a cancer-driven gene that is aberrantly activated in a variety of cancers, including gastric cancer, lung cancer, hepatocellular carcinoma, prostate cancer and colorectal cancer, and it facilitates tumorigenesis and metastasis [29–34]. The activation of MMP1 and RAS/MAPK signaling have been reported to be involved in the regulation of *ETV4* in cancer cells [33, 35, 36]. In addition, ETV4 is involved in the pancreatic cancer invasion and immune evasion by suppressing *PDCD4* mRNA translation [37]. However, the oncogenic function of *ETV4* in melanoma has not been systematically reported, and how *ETV4* expression is regulated in tumor initiation and progression is poorly understood.

In this work, we identified a new active enhancer (chr17: 59,785,798–59,787,315, hg38) in melanoma, named enh17 here according to its chromosomal location. We further explored its function in cell proliferation, migration, and tumor growth in melanoma. By integrative analysis of multi-omics data, we disclosed that the transcription factor STAT3, together with enh17, regulates the expression of *ETV4*. In the following series of functional experiments, we further demonstrated that *ETV4*, as the downstream target of enh17, plays a key role in cancer-related phenotypes, as well. For the first time, this work reports the oncogenic role of enh17 as a promising therapeutic target in melanoma.

## Materials and methods

### Quantification of eRNA

For skin cutaneous melanoma (SKCM) tumor and normal tissues from TCGA database, enhancer expression levels were obtained from The Cancer eRNA Atlas (TCeA) database (https://bioinformatics.mdanderson.org/Supplements/Super_Enhancer/TCEA_website/index.html) as RPKM (reads per kilobase per million). For RNA-seq raw data downloaded from the GEO database (GSE153592, GSE200217, and GSE35704), we used Tophat2 (v.2.0.13) to map fasta files to the enhancer reference genome, which was created based on the hg19 reference genome file and the chromosome location information of 65,000 enhancers annotated by Functional Annotation of the Mammalian Genome (FANTOM) (https://slidebase.binf.ku.dk/human_enhancers/) database. The key parameters include “-read-edit-dist 5 --read-mismatches 5 --read-gap-length 5 --mate-inner-dist 400 --mate-std-dev 400 --min-intron-length 10 --max-insertion-length 10 --max-deletion-length 9 --num-threads 3 --segment-mismatches 3”. The results were counted by htseq-count (v.0.11.1) for expression statistics, and the final expression values were converted to RPKM values, to maintain consistency with the results downloaded from TCeA. DESeq2 (v.1.38.3) was performed for differential expression analysis, with the criteria of fold change > 2.0 and *Padj* < 0.05 as the screening thresholds for differential enhancers.

### Copy number variation (CNV) analysis

The SKCM copy number profile were downloaded from the TCGA Genomic Characterization Center (http://gdac.broadinstitute.org/runs/analyses_2016_01_28/data/SKCM/20160128/), which includes the CNV information of 472 SKCM patients. The CNVs of chromosomal segments were estimated by The TCGA FIREHOSE project using the GISTIC2 method. The results were denoted as five levels: -2 (homozygous deletion), -1 (single copy deletion), 0 (diploid as normal), 1 (low-level copy number amplification) and 2 (high-level copy number amplification). The variant information files were filtered to screen out chromosomal segments with changes covering enhancer and high copy number amplification using the R package maftools (v.2.14.0) for visualization.

### Survival analysis

First, we matched the clinical information of SKCM patients in TCGA with the eRNA expression level matrix of SKCM patients in TCeA database according to the TCGA case ID. We then screened out the patients with enh17 expression in the top 25% and bottom 25%, and compared the survival time between these two groups. Using the survival (v.3.4.0) package in R, we evaluated the effect of the enh17 expression level on survival in SKCM patients. *P* value < 0.05 was deemed significant.

### Chromatin immunoprecipitation sequencing (ChIP–seq) data analysis

ChIP-seq data of fasta files were obtained from the GEO databases (A375 H3K4me1 ChIP-seq data: GSE75356; A375 H3K27ac ChIP-seq data: GSE99836; skin H3K4me1 ChIP-seq: GSE75356; skin H3K27ac ChIP-seq: GSE89129). Sequencing reads were mapped to the human genome reference hg38 using Bowtie2 (v.2.4.5). MACS2 (v.2.2.7) was employed to detect peaks from the ChIP-seq data. The analysis was conducted with default parameters and visualized via IGV.

### Cell lines and cell culture

A375 (1101HUM-PUMC000126) and A875 (1101HUM-PUMC000094) melanoma cell lines were purchased from the Institute of Basic Medical Science, Chinese Academy of Medical Science (Beijing, China). Cells were cultured in DMEM medium (Gibco, USA) with 10% fetal bovine serum (Gibco, USA) and incubated at 37 °C with 5% CO_2_. Cells were detached with 0.05% trypsin solution (Gibco, USA) and dispersed into single-cell suspension. Cells were passaged every 2–3 days for a maximum of 10 passages. The cell lines were confirmed by short tandem repeats (STR) analysis at Beijing Microread Genetics Co., Ltd. The cell lines were confirmed to be negative for mycoplasma determined by One-step Quickcolor Mycoplasma Detection Kit (Shanghai Yise Medical Technology Co., Ltd., China).

### enh17 knock out by CRISPR/Cas9

Two pair of guide RNAs (gRNAs) were designed using the CRISPR design website (crispr.mit.edu) and cloned into plasmids. Primers used in gRNA plasmid construction were listed in Additional file 2: Table S1. For each pair, one sgRNA was cloned into pU6- (BbsI) CBh-Cas9-T2A-mCherry (Addgene #64324), and the other into pSpCas9 (BB)-2 A-GFP (PX458) (Addgene #48138).

The plasmids with gRNAs were transfected into A375 cells using Lipofectamine 3000 (Thermo Fisher Scientific, USA), and single cells were sorted into 96-well plates using FACS (fluorescence activated cell sorting), which were simultaneously positive for both GFP (green fluorescent protein) and mCherry. For the single cell clones obtained, the successful knock out of enh17 was verified by Sanger sequencing.

### Cell transfection

For siRNAs or locked nucleic acids (LNAs) transfection, siRNAs were synthesized by JTS scientific (Wuhan, China), and LNAs were synthesized by Sangon Biotech (Shanghai, China). 2 × 10^5^ cells were plated and then transfected with specific siRNAs (40 nM) or LNAs (30 nM) using Lipofectamine 3000 (Invitrogen, USA) according to the manufacturer’s protocol. For enh17 knockdown, a mixture of LNA oligo 1, 2, and 3 was transfected into cells. The siRNA sequences and LNA oligos were listed in Additional file 2: Table S1.

### Cell viability assay

For cell proliferation assay, cell viability assay was performed using CCK-8 Cell Counting Kit (Vazyme, Nanjing, China), according to manufacturer’s instruction. Briefly, 4,000 cells were seeded in 96-well plates, 10 µl CCK-8 solution was added into each well 48 h after siRNA/LNA transfection, and the OD value were detected at a wavelength of 450 nm. For clonogenic assay, 700 cells were plated in 6-well plate and cultured for 10–14 days. Then, the cells were fixed with 4% paraformaldehyde (Beyotime, Shanghai, China), stained with 0.1% crystal violet solution (Solarbio, Beijing, China), and counted subsequently.

### 5-Ethynyl-2′-deoxyuridine (EdU) assay

EdU assay was performed using the Click-iT EdU Imaging Kit (Biorigin, Beijing, China) following the manufacturer’s instructions. Images were captured using an inverted fluorescence microscope (Mshot). Cell counts were performed using ImageJ. EdU-positive cells were calculated as EdU-stained cells/Hoechst 33,342-stained cells.

### Wound healing assay

5 × 10^5^ cells were seeded into each well of 6-well plates. 24 h later, a vertical wound was created by scratching the monolayer with a sterile 200 µl pipette tip in the middle of each well, and then the wells were washed three times with PBS to remove the floating cells. At 0 h and 24 h after wound induction, photographs were taken under microscope at the same location to monitor cell migration into the wounded area. The wound healing rate was calculated as (the difference between 0 h wound area and 24 h wound area)/0 h wound area.

### Cell migration assay

3 × 10^4^ cell suspension without fetal bovine serum (FBS) was aspirated into the upper layer of the Transwell chamber (Corning, USA) and the bottom chamber was supplemented with medium containing FBS. After incubation for 24 h, the non-migrating cells in the upper chamber were removed and the migrating cells in the bottom chamber were fixed with 4% paraformaldehyde for 30 min and then washed twice with PBS. The cells were stained with crystal violet solution for 30 min and washed three times with PBS. The cells of the upper layer were removed gently with a cotton swab and the number of migrated cells in each chamber was counted under a microscope.

### RNA extraction and qPCR

Total RNA was extracted from cultured cell lines using the RNeasy Plus Mini Kit (Qiagen, Germany) according to the manufacture’s protocol. A total of 1 µg of RNA was then reverse transcribed to a final volume of 20 µl using the High-Capacity cDNA Reverse Transcription Kit (Thermo Fisher Scientific, USA) according to the manufacturer’s protocol. 2 µl of cDNA product was performed for qPCR using SYBR Select Master Mix (Thermo Fisher Scientific, USA) following the manufacturer’s instructions for each reaction. The primer pairs were listed in Additional file 2: Table S1. The 2^−ΔΔCt^ method is used to calculate the fold-change values of gene expression.

### Western blot

Cell protein was extracted using Pierce RIPA buffer (Thermo Fisher Scientific, USA) for 30 min on ice, as recommended by the manufacturer. The protein concentration was determined by Pierce BCA Protein Assay Kit (Thermo Fisher Scientific, USA). 3 µg protein was separated in 4–12% NuPAGE Novex Bis-Tris gels (Invitrogen, USA) with 1 × NuPAGE™ MES SDS running buffer (Thermo Fisher Scientific, USA), then transferred onto a 0.45 μm polyvinylidene fluoride (PVDF) membrane by electrophoresis. After blocking with 5% non-fat dry milk for 1 h at RT, the primary antibodies for detecting ETV4 (dilution: 1:2000, Abcam, ab189826), GAPDH (dilution: 1:1000, Abcam, ab9484), β-actin (dilution: 1:30000, Sigma, A3584) were incubated with the membrane at 4 °C overnight. Then, the membrane was incubated with the second antibody (dilution:1:10000, HRP-labeled goat anti-rabbit IgG, Zhongshan Golden Bridge Bio-technology) for 2 h at room temperature. Visualization was performed using the High-sig ECL Western Blotting Substrate (Tanon, Shanghai, China) and the Tanon Chemiluminescence Imaging System.

**In vivo** **tumorigenicity assay**

BALB/c nude mice (female, 5 weeks old) were purchased from Beijing Vital River Laboratory Animal Technologies Co. Ltd. (Beijing, China) and randomly divided into three or four groups (five mice per group). These mice were housed under specific pathogen-free (SPF) conditions. A total of 2 × 10^6^ A375 cells (control), enh17 knockout cells (enh17^−/−^_1, enh17^−/−^_2), and enh17^−/−^-*ETV4* overexpression (enh17^−/−^_1-ETV4, enh17^−/−^_2-ETV4) suspended in 200 µl PBS and were individually inoculated into the right flank of the nude mice. Seven days after injection, the tumor size (tumor length and width) was measured twice a week using calipers. At the end of 25 days, the mice were euthanized, and both the tumor size and tumor weight were measured. The tumor volume was calculated as follows: length × width^2^ × 0.52. All the animal experiments were approved by the Beihang University Biomedical Ethics Committee (approval number: BM20220062; approved on March 4, 2022).

### Stable overexpression cell line construction

To generate *ETV4-*overexpression in the enh17^−/−^ cell lines, the amplified cDNA fragment of *ETV4* was cloned into the pCDH lentiviral vector (System Biosciences, USA), the empty vector (pCDH-puro vector) was used as a control. For lentiviral particles production, 5 × 10^6^ HEK 293T cells were seeded into 10 cm dishes at 24 h, then 12 µg lentiviral vectors of pCDH-ETV4 or pCDH-puro in combination with 8 ug pCMV-dR8.2 (Addgene, Cat.8455) and 4 µg VSVG (Addgene, Cat.8454) were transfected into HEK 293T cells using Lipofectamine 3000. Viruses were collected at 48 h after transfection, and then the supernatant was used to infect cells with Polybrene (8 µg/ml, Santa Cruz). At 48 h post infection, stable overexpression cells were selected with 1 µg/ml of puromycin for five days.

### RNA sequencing (RNA-seq) and single-cell RNA-seq (scRNA-seq) data analysis

The *STAT3* knockdown RNA-seq was obtained from GSE31534. The library preparation and sequencing of enh17 knockout RNA-seq were performed by Novogene. The quality of the clean reads was ascertained through FastQC and the clean reads were aligned to the hg38 genome using Bowtie2 (v.2.4.5). Low-quality reads and PCR duplicates were eliminated using SAMtools (v.1.7). For preprocessed and normalized RNA-seq data, we employed DESeq2 (v.1.38.3) for differential expression analysis. The criteria of fold change > 2.0 and *Padj* < 0.05 as a threshold for screening. Gene Set Enrichment Analysis (GSEA) was performed using the gseGO function in the R package clusterProfiler (v.4.6.2), based on the Gene Ontology (GO) database. The log_2_(fold change of all genes) was employed as the basis for gene ranking. Gene sets with a false discovery rate (FDR) < 0.05 were considered as significantly enriched.

For scRNA-seq, single-cell transcriptome data from SKCM patients with and without metastasis were collected from GSE72056 and GSE115978 (Additional file 2: Table S2). Seurat (v.2.3.0) was employed to process the scRNA-seq data. Various cell populations were identified through the FindClusters program using the HumanPrimaryCellAtlasData dataset. The FeaturePlot command assessed the *ETV4* expression across diverse cell types, utilizing default parameters. All functions were executed with default settings.

### High-throughput chromosome conformation capture (Hi-C) data analysis

The Hi-C data for the A375 cell line were obtained from GSE143678. The analysis was conducted using Juicer (v.2.13.07). The Hi-C outcomes were visualized by the Juicebox software at a resolution of 5 Mb. Criteria for enhancer-target pair screening include within the same Topologically Associated Domain (TAD) and an O/E interaction > 1.

### Luciferase reporter assay

For the construction of the pGL3-luciferase reporter plasmid, the promoter region of *ETV4* and the enh17 region were amplified from genomic DNA. The promoter region of *ETV4* was cloned into the pGL3-Basic plasmids and then the enh17 region was cloned upstream of the promoter region of *ETV4*, and the pGL3-luciferase reporter plasmids with the promoter region of *ETV4* were used as control. The *ETV4* promoter primers and enh17 primers were listed in Additional file 2: Table S1. Subsequently, 1.5 × 10^5^ HEK293T cells were seeded into each well of 24-well plate for 24 h, 500 ng pGL3-luciferase reporter plasmids together with 50 ng Renilla pRL-TK vector as internal control were transfected into each well using Lipofectamine 3000 according to the manufacturer’s protocol. Finally, luciferase activity was analyzed with the Dual-Luciferase Reporter Assay System (Promega, USA) according to the manufacturer’s protocol by a Clariostar Microplate Reader (BMG Labtech, Germany) 48 h after transfection. The ratios of Firefly/Renilla activities were calculated and expressed as relative luciferase activity.

### Cleavage under targets and tagmentation (CUT&Tag) assay

The CUT&Tag assay was performed using a Hyperactive® Universal CUT&Tag Assay Kit (Vazyme, Nanjing, China) following the manufacturer’s protocol. 1 × 10^5^ living cells were collected for library preparation. 2 µg of primary antibody of mouse monoclonal anti-Stat3 antibody (Cell Signaling, #9139) or H3K27ac antibody (Abcam, ab4729) and 1 µg of secondary antibody (Vazyme, Nanjing, China) were used. The prepared libraries were sequenced on the Illumina NovaSeq platform at Novogene, with the output of ~ 500 M 150 bp paired-end reads for each sample. The sequenced reads were qualified using FastQC. The paired-end sequencing reads were then aligned to hg38 using Bowtie2 (v.2.4.5). SAMtools (v.1.7) was used to eliminate PCR duplicates. SEACR (v.1.3) was used for peak calling. Visualization was performed using IGV.

### Statistical analysis

All statistical tests performed are indicated in the figure legends. GraphPad Prism (v.7.0) was used for statistical analysis. Unpaired two-sided Student’s *t*-test was used for statistical significance between two groups. More than two groups were determined with one-way or two-way analysis of variance (ANOVA). Quantitative data are shown as mean ± SD. *P* < 0.05 was considered as statistically significant.

## Results

### The identification of essential enhancers in melanoma

**Fig. 1:**
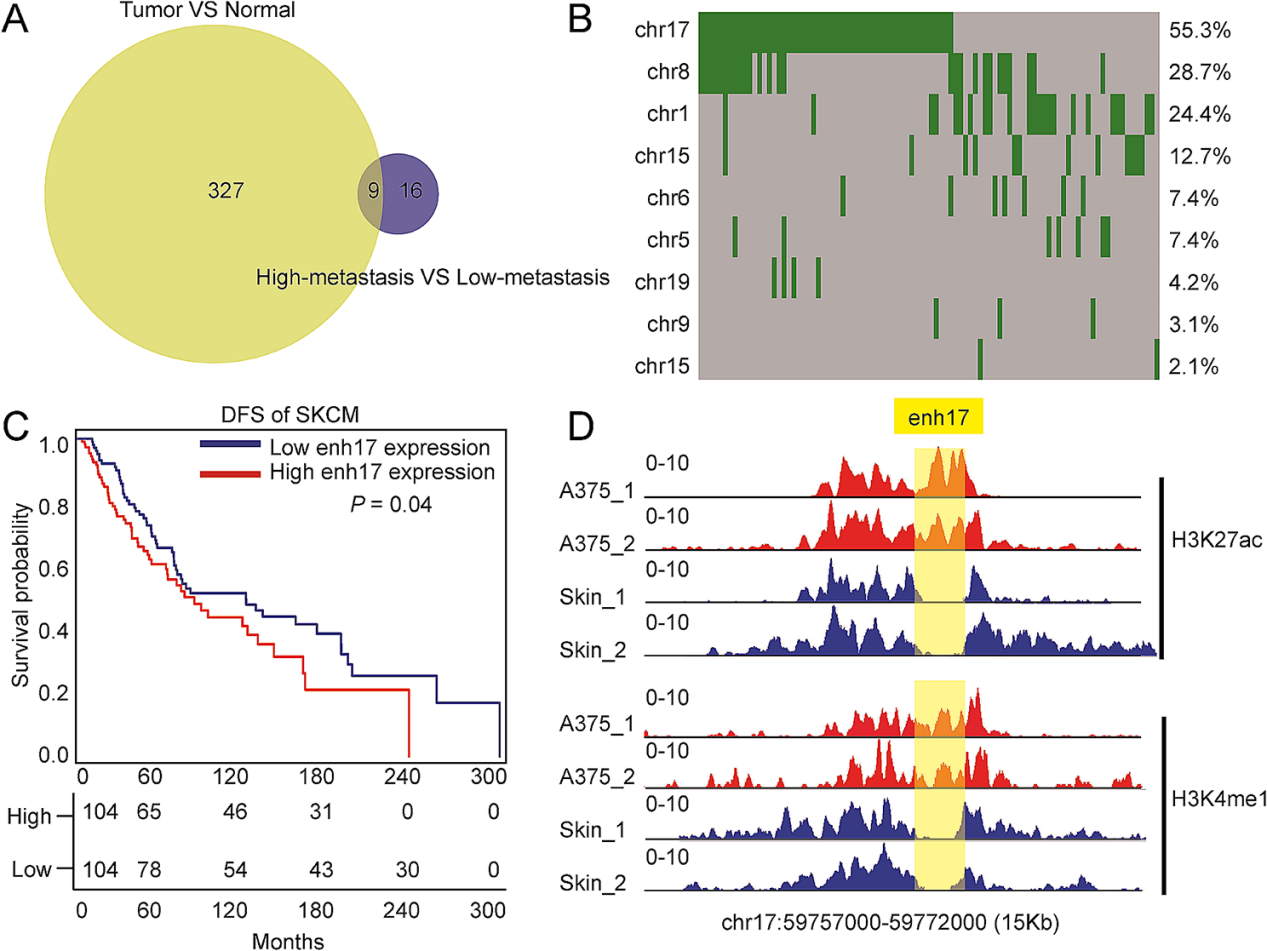
The identification of essential enhancers in melanoma. **A** The essential enhancers in melanoma identified by comparison of tumor/normal and high-metastatic/low-metastatic. **B** The heatmap shows the high level of copy number amplification of 9 candidate enhancers in SKCM patients. The green bar represents the high level of copy number amplification, and gray bar represents the absence of variation. The numbers on the right represent the proportion of patients with mutations out of a total of 94 patients. **C** Survival analysis shows the association between enh17 expression and DFS for SKCM. The numbers below represent the number of samples that survived at a different time point. **D** The ChIP-seq peaks of H3K27ac and H3K4me1 in A375 cell line and skin tissue. The duplications for each histone modification dataset are annotated as “_1” and “_2”, respectively, and the yellow part represents the genome region of enh17

eRNA expression is a widely used marker to characterize active enhancers. First, we identified 336 eRNAs upregulated in melanoma from the TCGA (Additional file 1: Fig. S1A). Next, we obtained transcriptome datasets from the GEO database (GSE153592, GSE200217, and GSE35704) and analyzed the eRNA expression differentiation related with melanoma invasion or metastasis (Additional file 1: Fig. S1B-D, Additional file 2: Table S2). GSE153592 includes RNA-seq data from 8 invasive and 2 non-invasive melanoma cell lines. GSE200217 includes RNA-seq data from 4 patients with melanoma brain metastasis (MBM) and 4 patients with extracranial melanoma metastasis (ECM), supposing MBM with higher metastasis [38]. For GSE35704, we analyzed RNA-seq data of two cell lines, HEMn and A2058, representing the normal stage and the metastatic stage of human melanoma, respectively [39]. A total of 25 enhancers were upregulated in three metastatic datasets (Additional file 1: Fig. S1E). Take the intersection of the above results, a total of 9 enhancers were characterized to be associated with melanoma (Fig. 1A). Since enhancers beneficial to cancer cells are more likely to be amplified, CNV analysis was performed for 472 melanoma patients from the TCGA dataset. There are 94 patients with high levels of copy number amplification, and the amplification range covered the above 9 enhancer fragments. Of note, enh17 is the most frequently amplified enhancer with the incidence of 55.3% among melanoma patients (Fig. 1B). Survival analysis showed that patients with higher enh17 expression had shorter disease-free survival (DFS) time (Fig. 1C). In addition, ChIP-seq data showed that the binding peaks of H3K4me1 and H3K27ac were enriched in the enh17 region, denoting that enh17 is active in the A375 melanoma cell line (Fig. 1D). These results indicate that enh17 is most likely to be essential in melanoma.

### enh17 deletion suppresses cell proliferation and migration of melanoma cells

**Fig. 2:**
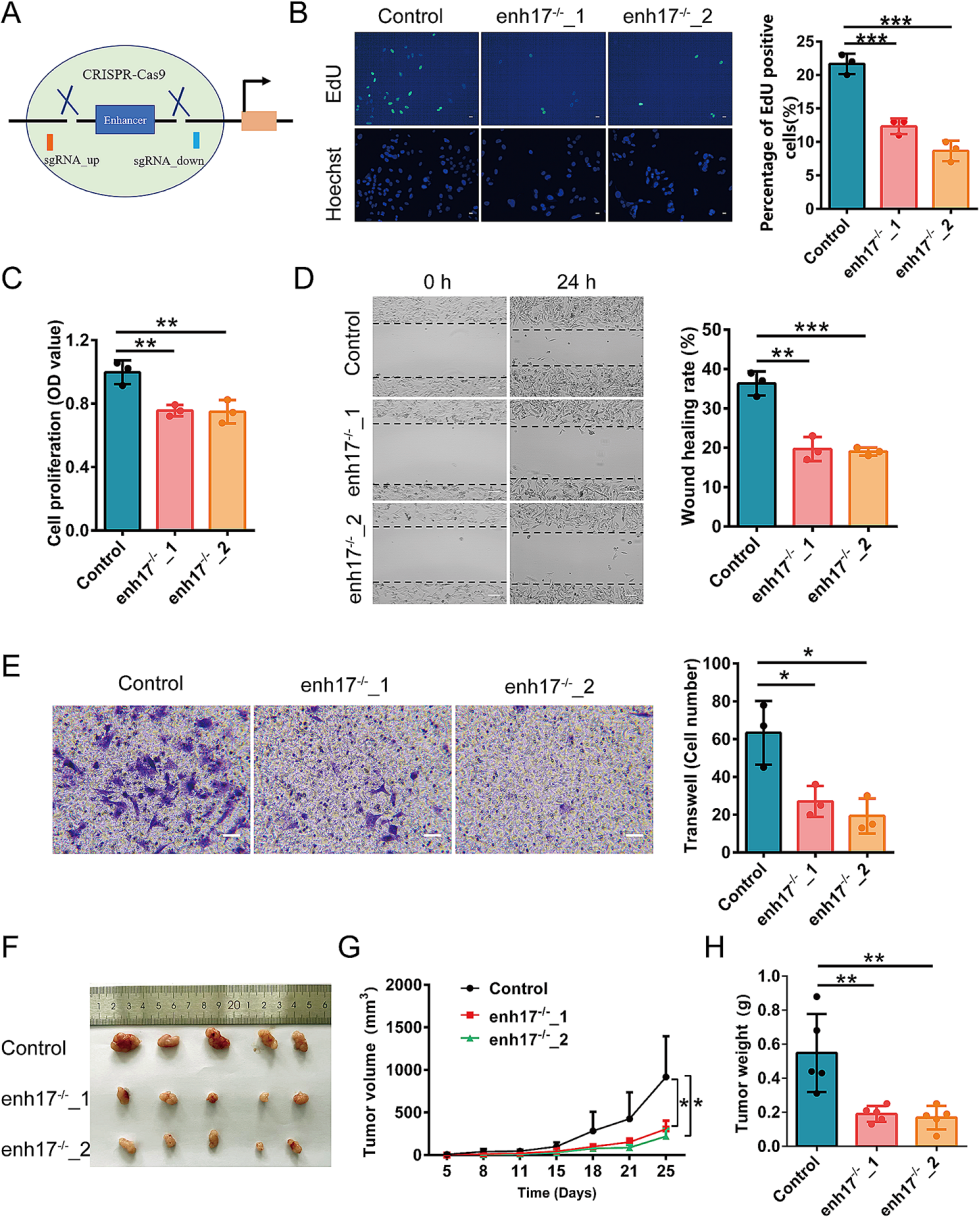
enh17 knockout inhibits the cell proliferation and migration of melanoma cells. **A** The illustration of the CRISPR/Cas9-mediated deletion of enh17. **B** EdU assay for enh17 knockout cells and the control. Left: Representative images with scale bar as 30 μm. Right: The statistical analyses of the ratio of EdU-positive cells were presented in graph. **C** CCK-8 assay for A375 cells with enh17 knockout and the control. **D** Wound healing assay for enh17 knockout cells and the control. Left: Representative images with scale bar as 100 μm. Right: Graph shows the quantification of the wound healing rate. **E** Transwell assay for enh17 knockout and the control. Left: The representative images with scale bar as 50 μm. Right: Graph shows the quantification of cells migrated to the lower layer of the membrane. **F-H** Xenograft experiments for enh17 knockout. The images of tumors (**F**), growth rate (**G**), and weight (**H**) of the xenograft tumor were shown (*n* = 5). enh17^−/−^_1 and enh17^−/−^_2: enh17 knockout by CRISPR/Cas9, A375 as control group. Results are presented as the mean ± SD, *n* = 3 in **B-E**, *n* = 5 in **F-H**. Results are analyzed using non-paired Student’s *t* test. *P < 0.05, **P < 0.01, ***P < 0.001

To explore the function of enh17 in melanoma cells, enh17 was firstly specifically knocked out by CRISPR/Cas9 technology using two pairs of gRNAs in A375 (Fig. 2A). Agarose gel electrophoresis and DNA sequencing confirmed that two enh17 knockout cell lines (enh17^−/−^_1, enh17^−/−^_2) were obtained (Additional file 1: Fig. S2A, B). Next, we investigated whether the deletion of enh17 diminish cancer-associated cellular phenotypes. EdU assay demonstrated that the proportion of EdU-positive cells in enh17 knockout cells was significantly reduced (Fig. 2B). CCK-8 assays also revealed that the knockout of enh17 significantly reduced the cell proliferation ability in A375 cells (Fig. 2C). Additionally, the knockout of enh17 showed less colony formation ability than the control cells (Additional file 1: Fig. S3). Furthermore, both wound healing assay and transwell assay showed that enh17 knockout led to significantly reduced cell migration ability (Fig. 2D, E). To confirm the effects of enh17 in tumorigenesis in vivo, tumorigenicity assay further showed that enh17 knockout could decrease xenograft growth, with a significant reduction in tumor size and weight compared to control group (Fig. 2F-H).

**Fig. 3:**
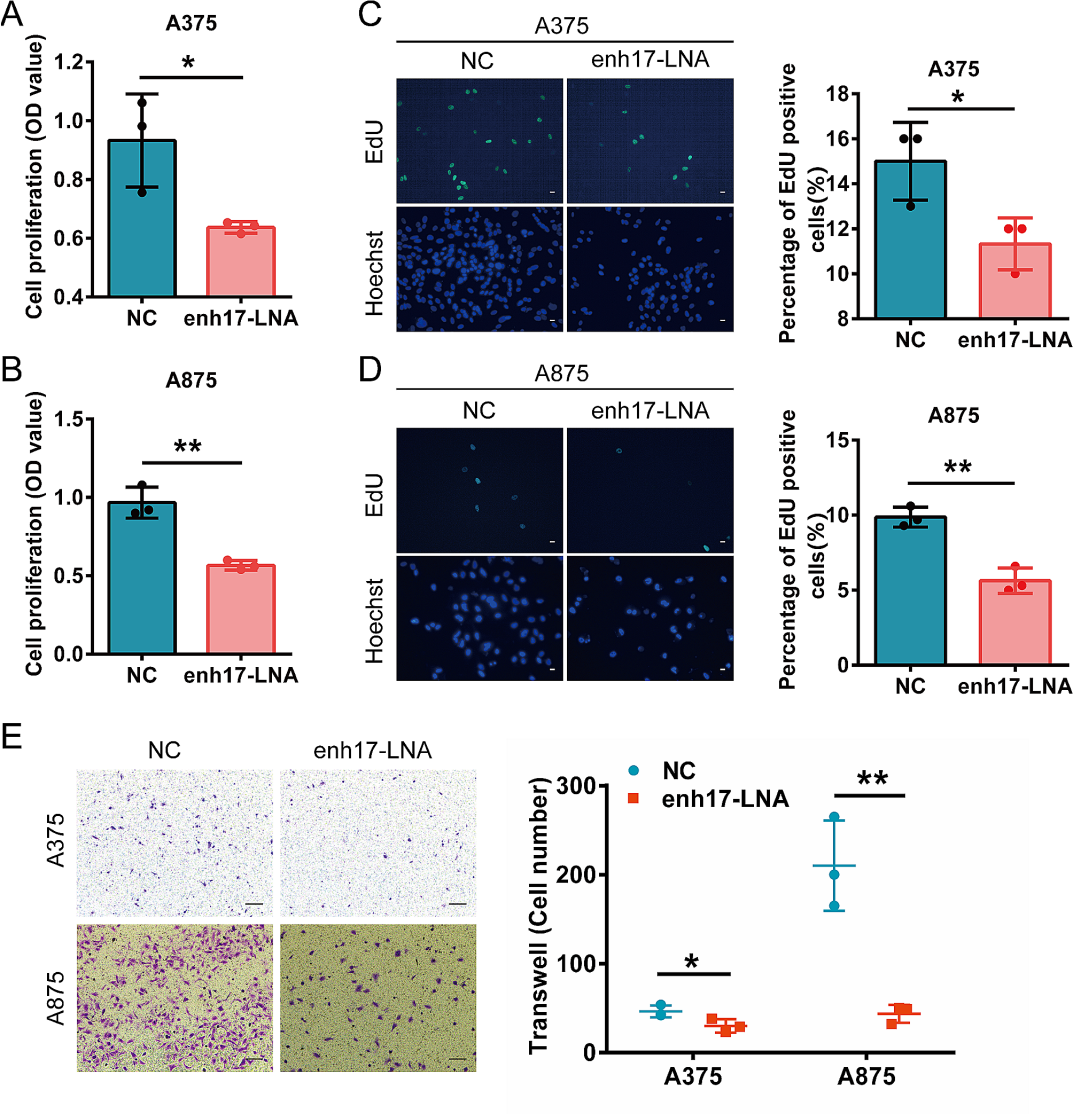
The inhibition of enh17 activity reduces cell proliferation and migration in both A375 and A875 cells. **A-B** CCK-8 assay for cell viability of A375 (**A**) and A875 (**B**) cells with enh17 knockdown. **C-D** EdU assay in A375 and A875 cells with enh17 knockdown. Left: Representative images in A375 (**C**) and A875 (**D**) with scale bar as 30 μm. Right: The statistical analyses of the ratio of EdU-positive cells in A375 (**C**) and A875 (**D**) were presented in graph. **E** Transwell assay for cell migration of A375 and A875 cells with enh17 knockdown. Left: The representative images with scale bar as 100 μm. Right: Graph shows the quantification of cells migrated to the lower layer of the membrane. All the data are presented as mean ± SD (*n* = 3). *P* values are calculated using non-paired Student’s *t* test. *P < 0.05, **P < 0.01

To further characterize the function of enh17 in melanoma, three LNAs to knock down enh17 were transfected into A375 and A875 cell lines, respectively. Consistent with enh17 knockout by CRISPR/Cas9, the cell proliferation was inhibited by LNAs in both A375 and A875 cell lines (Fig. 3A-D). Additionally, the transwell assay showed that the cell migration ability was also significantly reduced (Fig. 3E). Collectively, both in vitro and in vivo results suggest that enh17 play a key role in melanoma cell proliferation, migration, and tumor growth.

### *ETV4* regulates cell proliferation and migration in melanoma as a downstream target of enh17

**Fig. 4:**
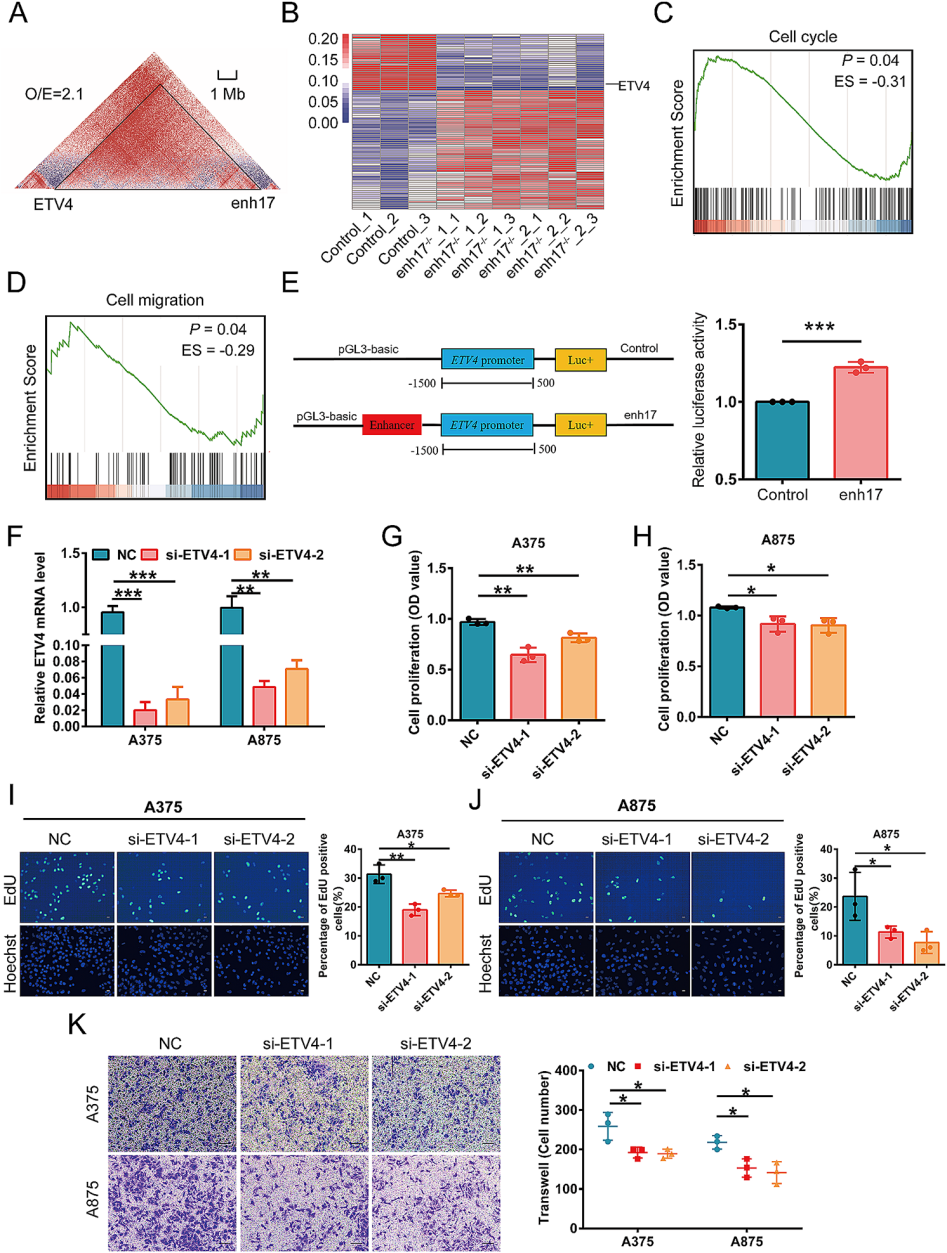
*ETV4* as the target gene regulated by enh17 and involve in cell proliferation and migration in melanoma. **A** Hi-C heatmap shows the genomic contact between enh17 and the promoter of *ETV4* at the chromosomal level. **B** HeatMap demonstrates the DEGs by analyzing the RNA-seq data between enh17^−/−^ and A375 control cells. **C-D** GSEA enrichment plots show that cell cycle (**C**) and cell migration (**D**) are negatively affected by enh17 knockout. **E** The luciferase activity was measured by luciferase reporter assay. Left: The illustration of the construction of the pGL3-luciferase reporter plasmids. Right: The relative luciferase activity was calculated by normalizing the Firefly luciferase activity with the Renilla luciferase activity. **F** The mRNA level of *ETV4* was determined by qPCR after *ETV4* knockdown in A375 and A875 cells by two siRNAs. **G-H** CCK-8 assay for cell viability of A375 (**G**) and A875 (**H**) cells with *ETV4* knockdown. **I-J** EdU assay in A375 and A875 cells with *ETV4* knockdown. Left: Representative images of A375 (**I**) and A875 (**J**) cells with scale bar as 30 μm. Right: The statistical analyses of the ratio of EdU-positive cells in A375 (**I**) and A875 (**J**) were presented in graph. **K** Transwell assay for cell migration of A375 and A875 cells with *ETV4* knockdown. Left: The representative images with scale bar as 100 μm. Right: Graph shows the quantification of cells migrated to the lower layer of the membrane. All the data are presented as mean ± SD (*n* = 3). *P* values are calculated using non-paired Student’s *t* test. *P < 0.05, **P < 0.01, ***P < 0.001

To investigate the underlying transcriptional regulation by enh17, Hi-C data from the A375 cell line was collected, and the genes within the same TAD with enh17 were identified as potential downstream targets of enh17 (Fig. 4A). RNA-seq analysis of enh17 knockout cell lines revealed differentially expressed genes (DEGs), and the genes that were downregulated when enh17 was knocked out were screened out as potential targets of enh17 (Fig. 4B). Combined the potential targets from Fig. 4A and B, *ETV4* is the most likely downstream target of enh17. Furthermore, Western blot results showed that *ETV4* protein levels were significantly decreased in enh17^−/−^ cell lines (Additional file 1: Fig. S4). In addition, we performed GSEA enrichment analysis on the RNA-seq results by using the clusterProfiler [40]. From the Gene Ontology database [41], the cell cycle (GO:0007049) and the cell migration (GO:0016477) were significantly enriched (Fig. 4C, D). Next, enh17 region was cloned into the pGL3-luciferase reporter plasmid with the promoter region of *ETV4* to validate whether *ETV4* is the target gene of enh17 (Fig. 4E, left), and the relative luciferase activity was significantly increased compared to control (Fig. 4E, right). These findings indicate that *ETV4* is the downstream target of enh17.

To evaluate the effect of *ETV4* on cell proliferation and migration, *ETV4* knockdown was performed using two siRNAs targeting *ETV4* in both A375 and A875 cells, a non-targeting siRNA as control. *ETV4* silencing was confirmed by qPCR (Fig. 4F). Consistent with enh17 knockout and knockdown results, *ETV4* knockdown significantly inhibited cell proliferation ability in both A375 and A875 cell lines as evaluated by the CCK-8 assay (Fig. 4G, H). EdU assay also demonstrated that the proportion of EdU-positive cells in *ETV4* downregulated cell lines was reduced (Fig. 4I, J). Furthermore, knockdown of *ETV4* resulted in a significant reduction in the ability of cells to migrate in both A375 and A875 cell lines (Fig. 4K). Together, we provided evidence to support the role of *ETV4*, whose expression is regulated by enh17, in the cell proliferation and migration of melanoma.

### Overexpression of *ETV4* restores the cellular phenotypes reduced by enh17 knockout in melanoma

**Fig. 5:**
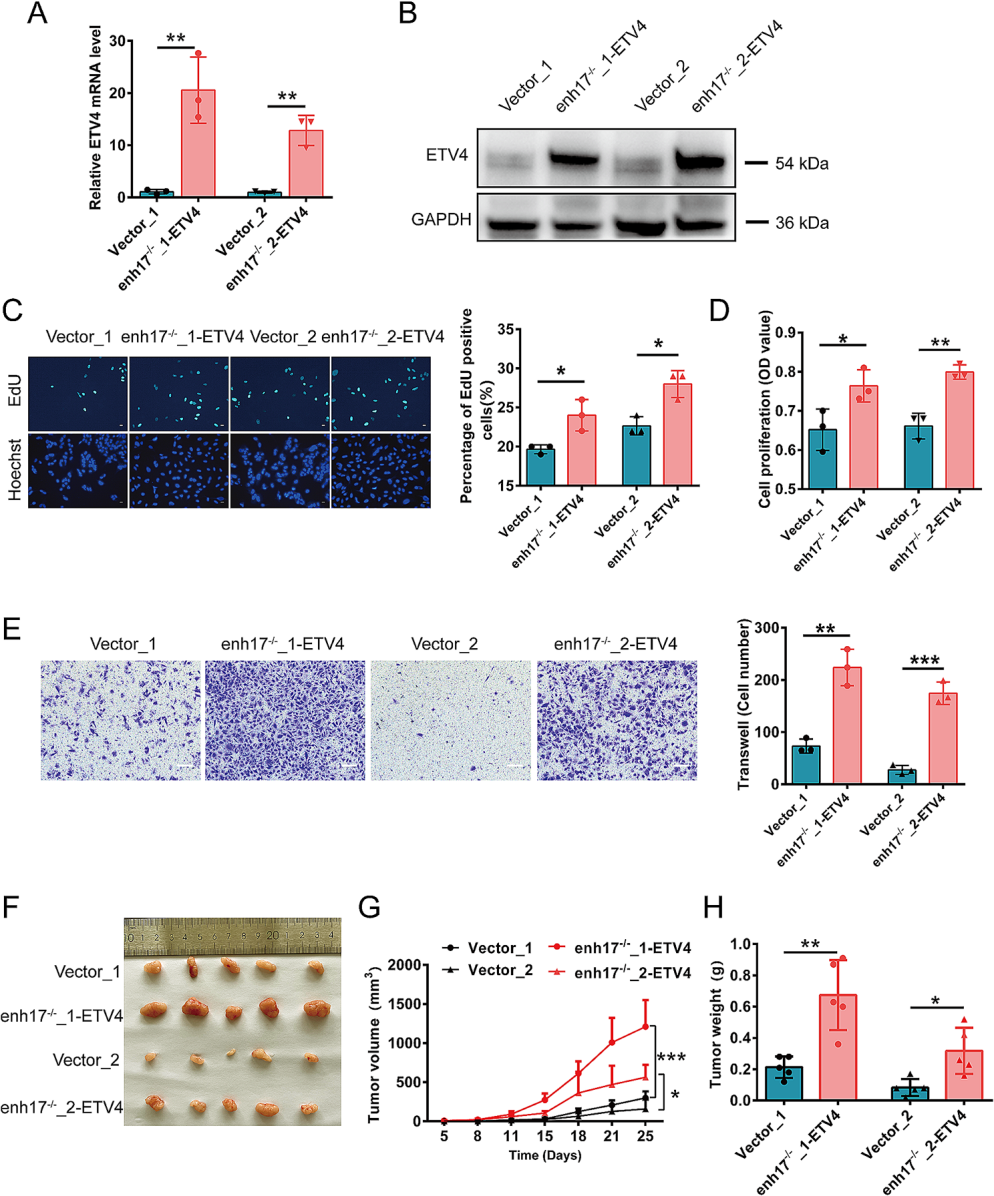
*ETV4* overexpression in enh17^−/−^cells restores enh17-regulated cellular phenotype. **A-B** qPCR and Western blot detection of *ETV4* RNA (**A**) and protein (**B**) abundance with *ETV4* overexpression in A375 enh17^−/−^ cells. **C** EdU assay in A375 enh17^−/−^ cells with *ETV4* overexpression. Left: Representative images with scale bar as 30 μm. Right: The statistical analyses of the ratio of EdU-positive cells were presented in graph. **D** CCK-8 assay for cell viability of A375 enh17^−/−^ cells with *ETV4* overexpression. **E** Transwell assay for cell migration of A375 enh17^−/−^ cells with *ETV4* overexpression. Left: The representative images with scale bar as 100 μm. Right: Graph shows the quantification of cells migrated to the lower layer of the membrane. **F-H** Xenograft experiments for *ETV4* overexpression in A375 enh17^−/−^ cells. The images of tumor (**F**, growth rate (**G**), and weight (**H**) of the xenograft tumor were shown (*n* = 5). “enh17^−/−^_1-ETV4”and “enh17^−/−^_2-ETV4” represents enh17^−/−^_1 and enh17^−/−^_2 cells infected by lentiviral *ETV4*-overexpression vector, respectively, while “Vector_1”and “Vector_2” represents enh17^−/−^_1 and enh17^−/−^_2 cells infected by lentiviral empty pCDH vector, used as control. Results are presented as the mean ± SD, *n* = 3 in **A-E**, *n* = 5 in **F-H**. *P* values are calculated using non-paired Student’s *t* test. *P < 0.05, **P < 0.01, ***P < 0.001

To validate *ETV4* is the downstream target gene of enh17 involved in cell proliferation, migration, and tumor growth, the overexpression of *ETV4* using a lentiviral vector was performed in two enh17^−/−^ cell lines. The qPCR and Western blot results showed that the transcription and protein levels of *ETV4* were significantly increased compared to the control groups, respectively. The results confirmed that the cell lines were successfully constructed to stably overexpress *ETV4* (Fig. 5A, B). Both EdU and CCK-8 assay showed that *ETV4* overexpression significantly restored the proliferation ability drop by enh17^−/−^ in A375 cells (Fig. 5C, D). The transwell assay showed that the migration ability of A375 enh17^−/−^ cells was restored by *ETV4* overexpression, as well (Fig. 5E). Additionally, in vivo tumorigenicity assay showed that *ETV4* overexpression rescued the xenograft growth. Both tumor volume and tumor weight were significantly increased compared to the control groups (Fig. 5F-H). These results above further validated that *ETV4* was the target gene of enh17, which is involved in the regulation of cell proliferation, migration, and tumor growth in melanoma.

### The transcription factor STAT3 is involved in the transcriptional regulation of *ETV4* by enh17

**Fig. 6:**
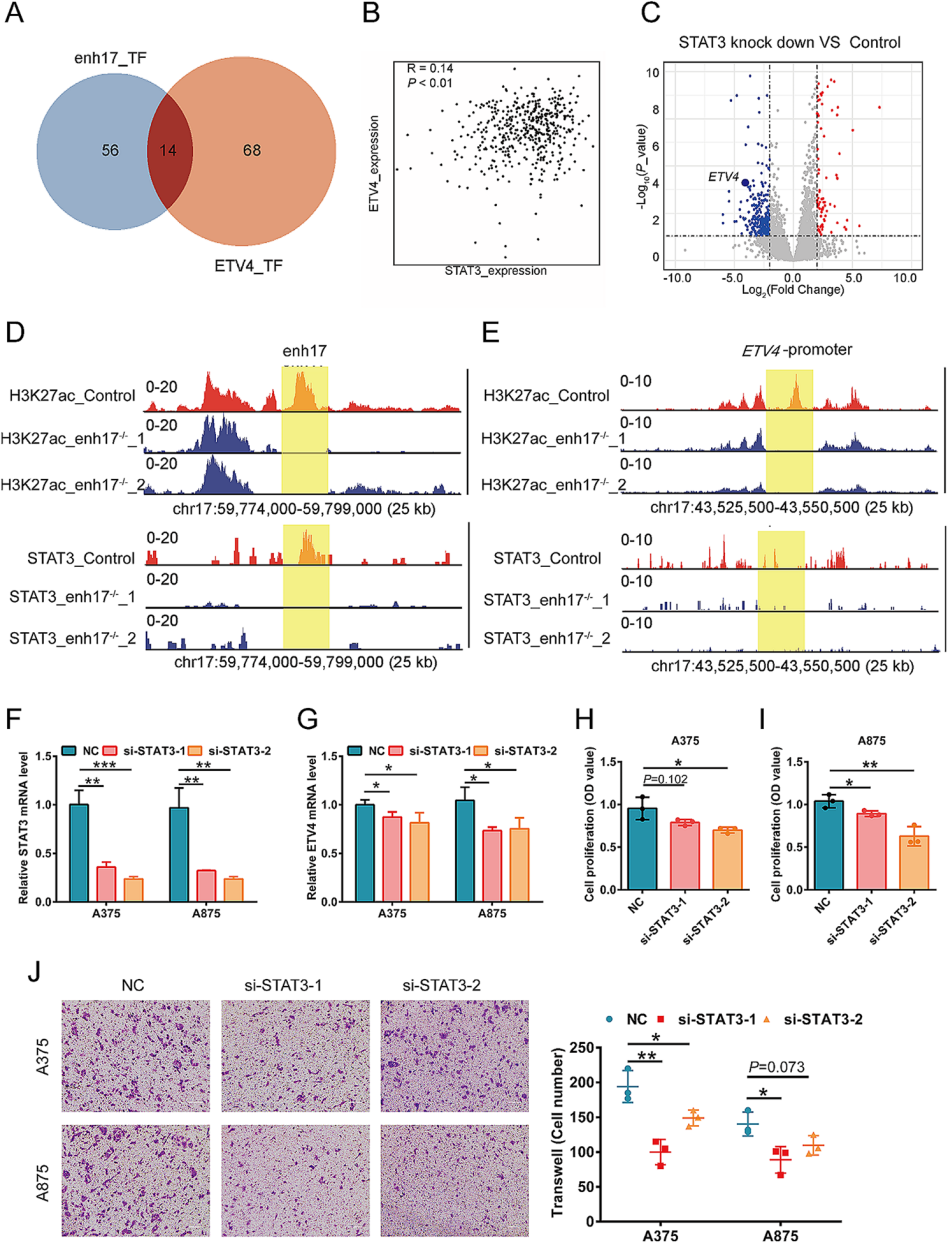
The transcription factor STAT3 binds to enh17 to regulate the transcription of *ETV4*. **A** Venn diagram shows the transcription factors that bind to both enh17 and the *ETV4* promoter regions. **B** The expression correlation between *ETV4* and *STAT3* in the TCGA database. **C** The volcano plot shows the DEGs after STAT3 knockdown, including *ETV4*. **D-E** IGV analysis of CUT&Tag coverage, H3K27ac and STAT3 peaks at the enh17 (**D**) and the *ETV4* promoter regions (**E**) with the indicated scale in A375 control and enh17 knockout cells determined by CUT&Tag. The enh17 genomic region and *ETV4* promoter regions are highlighted in yellow. **F** The knockdown efficiency of siRNAs targeting *STAT3* was determined by qPCR in A375 and A875 cells. **G** The mRNA level of *ETV4* was determined by qPCR after *STAT3* knockdown in A375 and A875 cells. **H-I** CCK-8 assay for cell viability of A375 (**H**) and A875 (**I**) cells with *STAT3* knockdown. **J** Transwell assay for cell migration of A375 and A875 cells with *STAT3* knockdown. Left: The representative images with scale bar as 100 μm. Right: Graph shows the quantification of cells migrated to the lower layer of the membrane. All the data are presented as mean ± SD (*n* = 3 in **F-J**). *P* values are calculated using non-paired Student’s *t* test. *P < 0.05, **P < 0.01, ***P < 0.001

Mechanistically, enhancers are occupied by TFs and stimulate the transcription of target genes, so the identification of key TFs is of great importance for enhancer research. To identify TFs, we analyzed the TFs that bind to both enh17 region and *ETV4* core promoter region based on the binding motifs reported by Human transcription factors database (HumanTFDB) [42]. 56 TFs bind to enh17 region and 68 TFs bind to *ETV4* core promoter region, there are 14 overlapping TFs (Fig. 6A). Subsequently, the correlation between *ETV4* and TFs was analyzed using the mRNA expression data from TCGA database. The results showed that *ETV4* had the significant correlation with the transcription factor STAT3 (Fig. 6B). RNA-seq data of A375 cells from the NCBI GEO database also showed that the expression of *ETV4* was greatly decreased when the expression of STAT3 was downregulated (Fig. 6C). Furthermore, CUT&Tag assay was performed to validate the binding profiles of STAT3 within enh17 and the promoters of *ETV4*. The knockout of enh17 attenuated STAT3 binding to both enh17 and *ETV4* promoters (Fig. 6D, E). To further confirm the transcription regulation axis enh17/STAT3/*ETV4*, *STAT3* knockdown experiments were performed using two siRNAs in both A375 and A875 cells, with a non-targeting siRNA as control. *STAT3* silencing was confirmed by qPCR (Fig. 6F). When *STAT3* was knocked down, the expression level of *ETV4* was correspondingly decreased (Fig. 6G). Furthermore, *STAT3* knockdown inhibited cell proliferation and migration ability in both A375 and A875 cells (Fig. 6H-J). Collectively, these data demonstrate that transcription factor STAT3 is involved in the transcriptional regulation of *ETV4* by enh17.

## Discussion

Melanoma is a typical cutaneous malignancy that originates from melanocytes. It is the most aggressive skin carcinoma with an overall mortality rate of more than 10%, and it is associated with the vast majority of skin cancer-related deaths [43, 44]. To develop effective new strategies for the treatment of melanoma, the elucidation of the molecular mechanisms underlying the development of melanoma and the identification of effective therapeutic targets are of paramount importance. In this study, we newly characterized one enhancer element that was responsible for the regulation on *ETV4* transcription in melanoma cells. Mechanistically, this enhancer is bound by the key transcriptional regulator STAT3 and promotes *ETV4* expression, ultimately leading to cancer-related phenotypes.

As one of the major *cis*-regulatory elements of genes, enhancers can positively regulate gene expression through long-range interaction with target genes, independent of genomic distance and orientation [45]. Due to the presence of typical histone marks and transcriptional co-activators, an increasing number of enhancers have been characterized in the genome [23]. Previous studies have found that the disease risk-associated SNPs identified by GWAS tend to be located in the non-coding regions, especially the enhancer regions [10, 46–48]. Small molecule inhibitors that specifically target non-coding regulatory sequences may have greater potential in the treatment of disease [49–51]. Currently, enhancer research is focused on screening and identifying active enhancers in the genome. Few enhancers have been studied for their functional role in a variety of tumors. In this study, we identified an active enhancer (enh17) by integrating multi-omics data, and further experimental validation revealed that enh17 was involved in the cell proliferation, migration, and growth of melanoma cells. Therefore, the biological significances and molecular mechanisms of enh17 in transcriptional regulation deserves in-depth investigation.

Enhancers can achieve their functional pleiotropy by modulating their target genes [52]. In hepatocellular carcinoma, an *PRMT7* enhancer was found to bind to the transcription factor HNF4A to promote the expression of the host gene *PRMT7*, which ultimately contributes to HCC pathogenesis [53]. MLL3, a histone H3K4 methyltransferase, which is frequently mutated in cancer. In breast cancer, Zheng et al. identified an enhancer associated with *MLL3*, and *MLL3* deletion promoted cell migration and resulted in reduced activity of an enhancer approximately 7 kb upstream of *TNS3*, and further inhibition of this enhancer activity by the dCas9 KRAB system decreased expression of the target gene *TNS3* [54]. Studying the regulatory mechanisms of enhancers and linking enhancers to target genes can help identify novel diagnostic and therapeutic targets. As an important member of the PEA3 subgroup, *ETV4* has been found to be overexpressed in multiple cancers and induce cell growth, invasion, and migration, suggesting its essential role in tumor progression [31, 32, 55]. In non-small cell lung cancer and breast cancer, *ETV4* was found to promote the migration and metastasis by transactivating MMPs [31, 56]. In pancreatic ductal adenocarcinoma (PDAC), *ETV4* overexpression promoted cell growth and facilitated cell cycle progression through transcriptional regulation of cyclin D1 [57]. Other work has reported that *ETV4* promotes PDAC cell migration and invasion by inducing EMT [27]. However, no study has yet demonstrated whether there is an enhancer region associated with *ETV4* that affects the expression or function of *ETV4* as well as contributes to cancer risk or progression. In the present study, we investigated for the first time that *ETV4* as a target of the enh17, based on multi-omics data analysis and experimental dissection. Both *ETV4* knockdown and rescue experiments provide further evidence that *ETV4* is involved in the regulation of cell proliferation and migration downstream of enh17 in melanoma cells. As described in Fig. 4B, there are a large set of genes up- or down-modulated when enh17 was knocked out by CRISPR/Cas9. In addition to *ETV4*, there should be more target genes directly or indirectly regulated by enh17 during melanoma progression. The observation is consistent with the statistics that one enhancer regulates multiple target genes (~ 2.4 on average) [5]. The target genes may function cooperatively to perform the pleiotropic of enh17 in melanoma. However, only *ETV4* was selected for further study, as only *ETV4* was in the TAD structure with enh17 among the DEGs in Fig. 4A. But whether these DEGs are involved in the function of enh17 and whether they are downstream targets of *ETV4* remain to be studied further. In addition, we also noticed that *ETV4* expression significantly increased in the metastatic tumor comparing to the primary tumor from TCGA data (Additional file 1: Fig. S5A), and the expression level of *ETV4* in metastatic cancer cells is higher than that in primary cancer cells from scRNA-seq data (Additional file 1: Fig. S5B). More studies should focus on exploring *ETV4* as a prognostic marker for melanoma and testing its potential value in melanoma treatment in the future.

STAT3, as a member of the Janus kinase-signal transducers and activators of transcription (JAK-STAT) family of proteins, is a cytokine-responsive transcription factor that responds to several cytokines and growth factors, including interleukin-6 (IL-6), oncostatin-M (OSM), and epidermal growth factor (EGF) [58]. In tumor initiation and progression, STAT3 acts as either tumor facilitator [59, 60] or tumor suppressor [61–65], playing a critical role in regulating cell proliferation, migration, apoptosis, and survival. In the present study, STAT3, as a transcription factor, binds to enh17 to regulate the expression of *ETV4*, thereby inducing cell proliferation and migration in melanoma cells. However, it is also possible that other transcription factors might be involved in regulating *ETV4* expression, as most enhancers contain multiple transcription factor binding sites [66]. The possibly mechanisms of *ETV4* are warranted to be further investigated in the future.

**Fig. 7:**
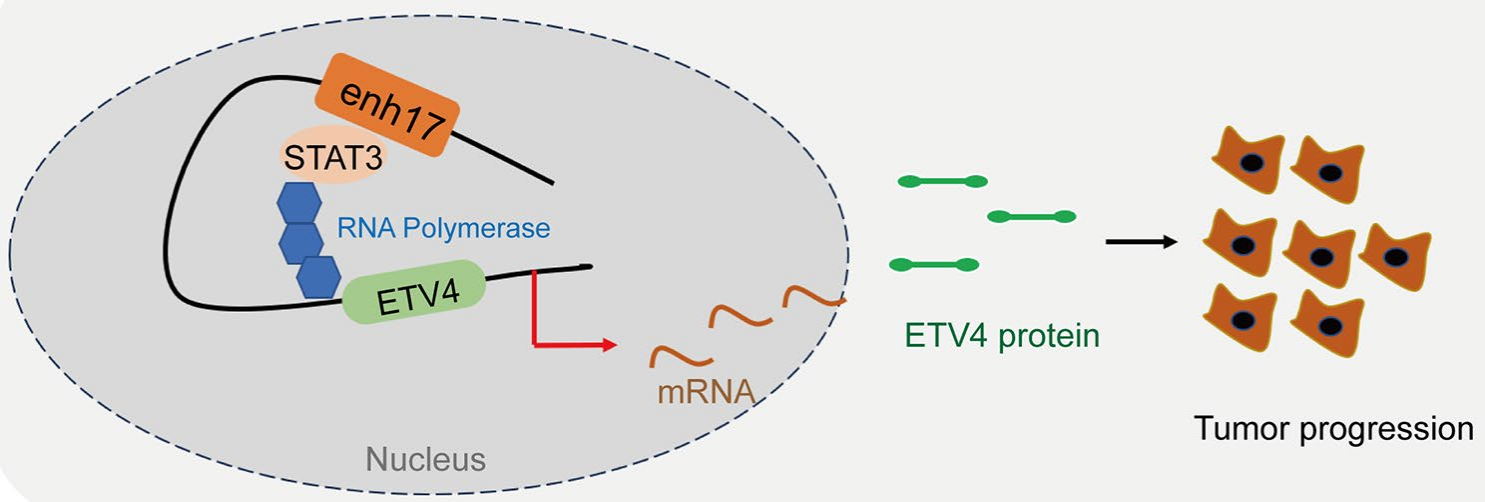
The model of transcriptional regulation by enh17 in melanoma. enh17 interacts with the transcription factor STAT3 and promotes *ETV4* transcription, promoting tumor progression

Conclusively, a specific *ETV4*-associated enhancer was identified in melanoma, and its causal role in cancer-related phenotypes was demonstrated. Transcription factor STAT3 was found to bind to the enh17 region to promote the expression of *ETV4*, which ultimately contributes to promoting tumor progression in melanoma (Fig. 7). This work sheds light on the regulatory mechanism and function of enh17 in melanoma, *ETV4*-enh17 represents a promising potential target for the prevention/ treatment of melanoma.

## Conclusions

The present findings suggest that enh17 plays an oncogenic role and promotes tumor progression in melanoma, and its transcriptional regulatory mechanisms were fully elucidated, which may open a promising window for melanoma prevention and treatment.

### Electronic supplementary material

Below is the link to the electronic supplementary material.

Supplementary Material 1

Supplementary Material 2

## Data Availability

The raw sequence data of RNA-seq and CUT&Tag reported in this paper have been deposited in the China National Center for Bioinformation (BioProject: PRJCA021718). The data and codes that supporting the findings of this study can be obtained from the corresponding authors upon reasonable request.

## Abbreviations

CNV: Copy number variation
ChIP-seq: Chromatin immunoprecipitation sequencing
CCK8: Cell Counting Kit-8
CUT&Tag: Cleavage under targets and tagmentation
DEGs: Differentially expressed genes
EdU: 5-Ethynyl-2′-deoxyuridine
EMT: Epithelial-mesenchymal transition
EGF: Epidermal growth factor
eRNAs: Enhancer RNAs
FBS: Fetal bovine serum
GEO: Gene expression omnibus
GSEA: Gene set enrichment analysis
GWAS: Genome-wide association study
H3K27ac: H3 lysine 27 acetylation
H3K4me1: H3 lysine 4 mono-methylation
Hi-C: High-throughput chromosome conformation capture
HumanTFDB: Human transcription factors database
IL-6: Interleukin-6
LNAs: Locked nucleic acids
OSM: Oncostatin-M
PDAC: Pancreatic ductal adenocarcinoma
RNA-seq: RNA-sequencing
SNPs: Single nucleotide polymorphisms
SKCM: Skin cutaneous melanoma
TCeA: The cancer eRNA atlas
TAD: Topologically associated domain
TFs: Transcription factors
