# Supplementary material for: An oncogenic enhancer promotes melanoma progression via regulating ETV4 expression
Source: J Transl Med. 2024 Jun 7;22:547. doi: 10.1186/s12967-024-05356-8 (PMC11157841; doi:10.1186/s12967-024-05356-8)
Supplement: Supplementary file 1 — Supplementary Material 1 [file 12967_2024_5356_MOESM1_ESM.docx]

**
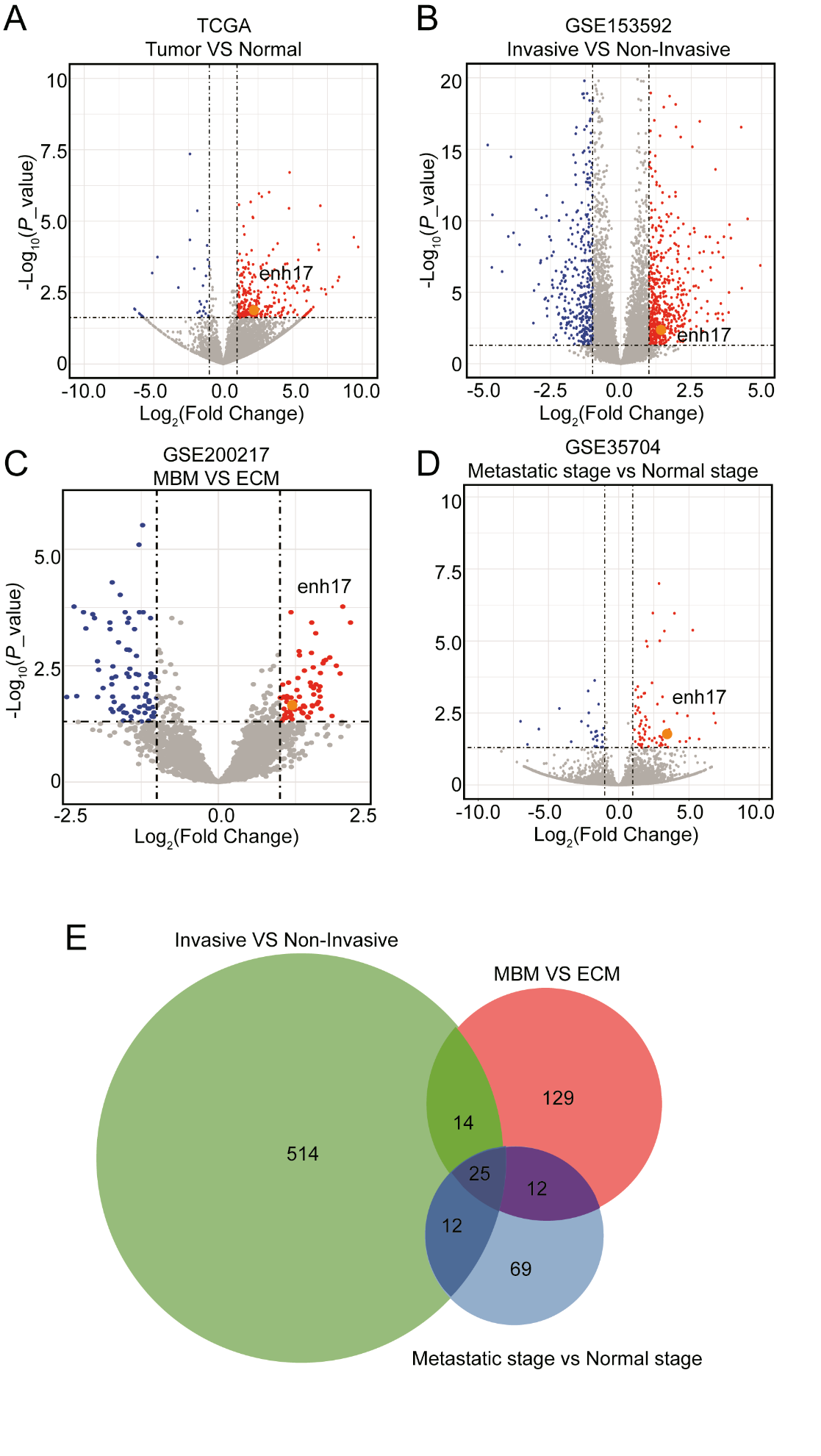
**

**Fig. S1 The differential expression analysis of eRNA in melanoma**. **A** Volcano plot of differentially expressed enhancers between tumor and normal. The red dots in the plot represent upregulated enhancers, the blue dots represent downregulated enhancers in tumor (fold change > 1.5, *Padj* < 0.05). Data were collected from TCGA. **B-D** Volcano plot of differentially expressed enhancers between high-metastasis and low-metastasis. The red dots in the plot represent upregulated enhancers, while the blue dots represent downregulated enhancers in metastasis (fold change > 1.5, *Padj* < 0.05). Data were collected from GSE153592 **(B)**, GSE200217 **(C)**, and GSE35704 **(D)**. **E** The Venn diagram presents the results of the above three datasets (**B-D**).

**
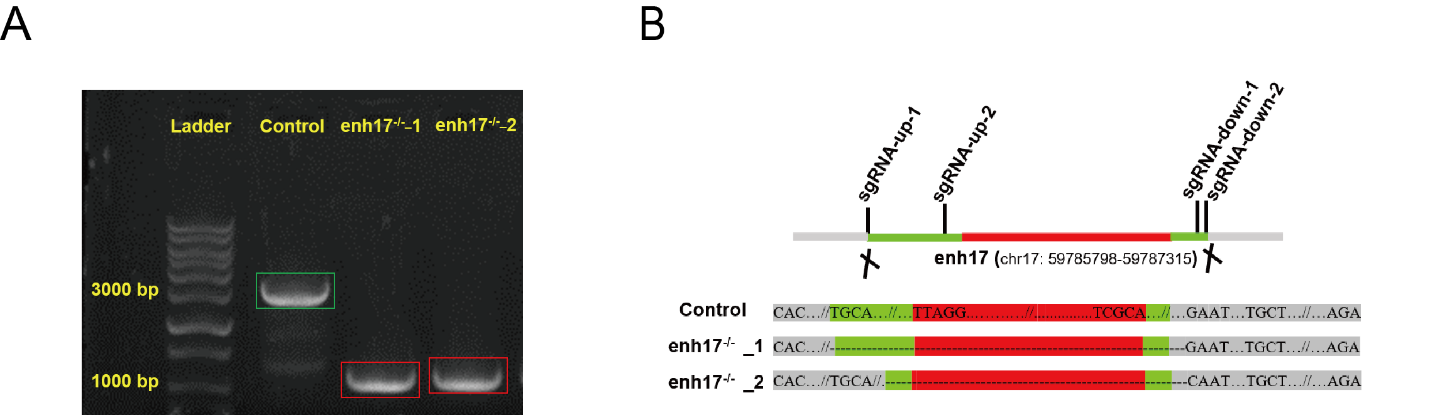
**

**Fig. S2** **enh17 was successfully knocked out**. **A** The agarose gel electrophoresis shows the size of the PCR products. **B** The Sanger sequencing results confirm the genomic region of enh17 was deleted as expected.


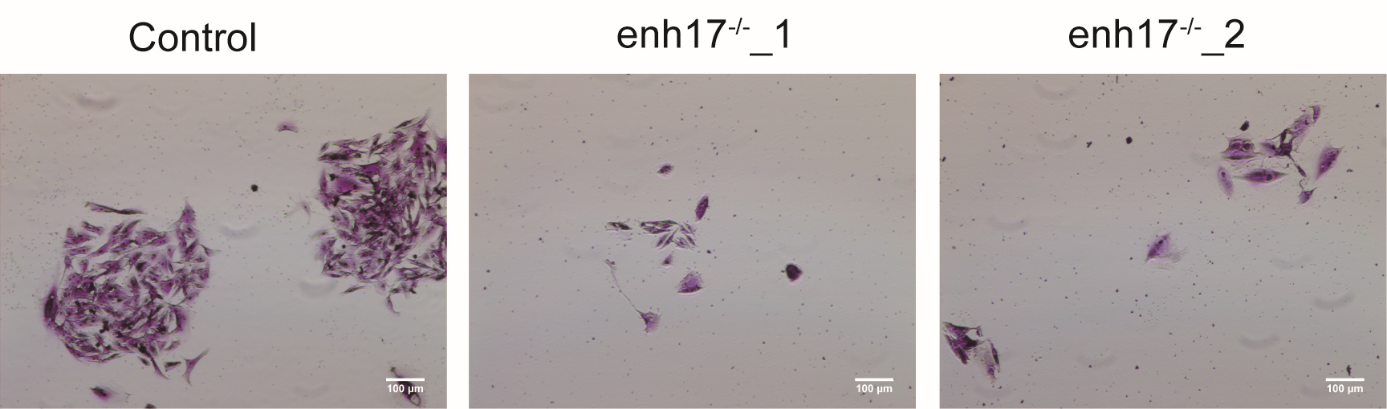


**Fig. S3 enh17 knockout inhibits the clonogenic ability of A375 cells.**

**
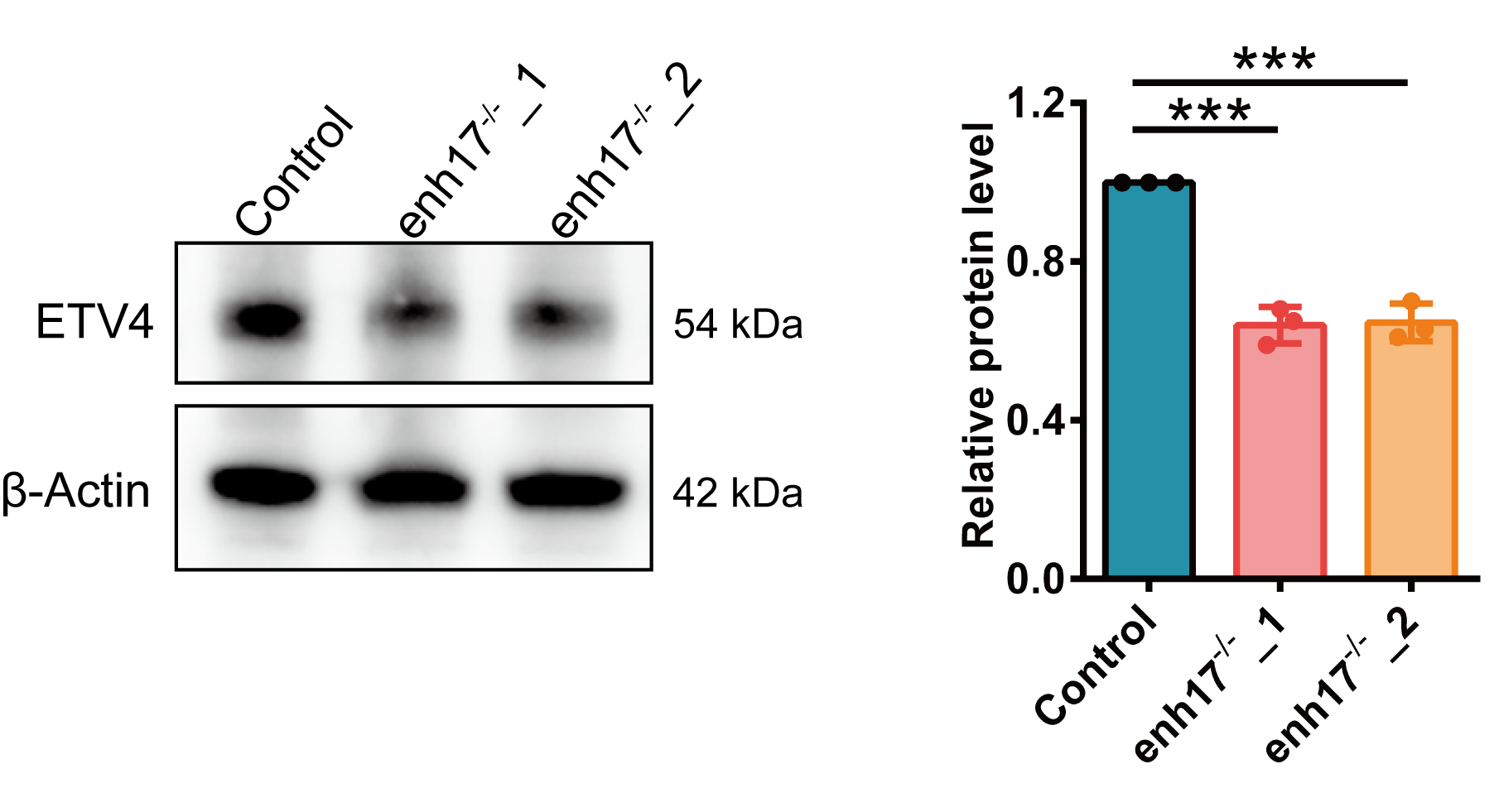
**

**Fig. S4 *ETV4* protein level is significantly decreased in enh17 knockout cell lines.** Representative result (left) and relative protein level (right) are shown. The data are presented as mean ± SD (*n* = 3). *P* values are calculated using non-paired Student’s *t* test. ****P* < 0.001.


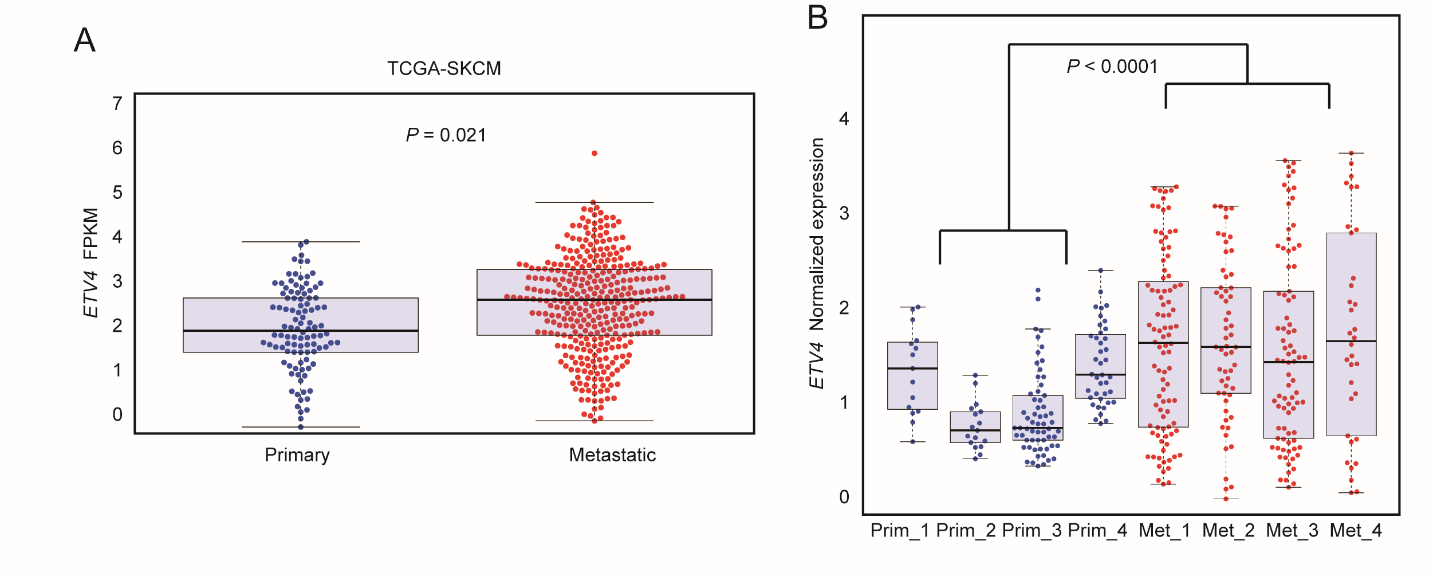


**Fig. S5 The expression of *ETV4* is significantly increased in the metastatic tissue*.* A** The expression level of *ETV4* in metastatic and primary SKCM tissues from TCGA data. **B** The expression level of *ETV4* in malignant cells from metastatic and primary tissues of SKCM patients. scRNA-seq data were collected from GSE72056 and GSE115978.
