# Supplementary material for: An oncogenic enhancer promotes melanoma progression via regulating ETV4 expression
Source: J Transl Med. 2024 Jun 7;22:547. doi: 10.1186/s12967-024-05356-8 (PMC11157841; doi:10.1186/s12967-024-05356-8)
Supplement: Supplementary file 2 — Supplementary Material 2 [file 12967_2024_5356_MOESM2_ESM.doc]

**Supplemental Table S1. Primers used in this study.**

| **Name** | **Sequences** |  |
| --- | --- | --- |
| Primers used for sgRNA plasmid construction | | |
| gRNA-1-F | 5′-GCUAAUUGAUAUGAAAAUAG-3′ |  |
| gRNA-1-R | 5′-GAUGCAAGCUGUUAAAUGAA-3′ |  |
| gRNA-2-F | 5′-GAUUCUU UCUCACAGUGUU-3′ |  |
| gRNA-2-R | 5′-CUGUUAAAUGAAUGGCCAG-3′ |  |
| Primers used for *ETV4* knock down | | |
| si-ETV4-1 | 5′-GCGUUGUCCCUGAGAAAUUdTdT-3′ |  |
| si-ETV4-2 | 5′-CCCUCUUCUCUUUGGCCUUdTdT-3′ |  |
| si-STAT3-1 | 5′-GGAAACAACCAGUCAGUGAdTdT-3′ |  |
| si-STAT3-2 | 5′-GGCGUCCAGUUCACUACUAAAdTdT-3′ |  |
| Primers used for enh17 knock down | | |
| LNA-1 | (lT)*(lT)*(lA)*C*T*G*C*A*A*A*G*C*T*T*A*A*(lT)*(lG)*(lC) | **l** represents Locked-nucleic acid; ***** represents Thio-modification |
| LNA-2 | (lT)*(lA)*(lA)*A*C*T*C*A*A*A*T*G*A*T*C*A*(lA)*(lA)*(lC) |
| LNA-3 | (lT)*(lT)*(lG)*T*G*A*T*A*A*A*A*T*C*A*T*G*(lA)*(lG)*(lC) |
| NC | (lA)*(lC)*(lC)*T*T*G*A*C*G*T*A*C*G*(lT)*(lT)*(lG) |
| Primers used for qPCR | | |
| ETV4-F | 5′-GAAGGAGACATCAAGCAGGAA-3′ |  |
| ETV4-R | 5′-AGCAAGGCCACCAGAAAT-3′ |  |
| STAT3-F | 5′-GAGAAGGACATCAGCGGTAAG-3′ |  |
| STAT3-R | 5′-CAGTGGAGACACCAGGATATTG-3′ |  |
| β-actin-F | 5′-ATTGGCAATGAGCGGTTC-3′ |  |
| β-actin-R | 5′-CGTGGATGCCACAGGACT-3′ |  |
| Primers used for pGL3-luciferase reporter plasmid construction | | |
| ETV4-P-F | 5′-ACAGGCCCAAAGAGCTTACA-3′ |  |
| ETV4-P-R | 5′-GTTTCTGCTTTCTGCAGCCC-3′ |  |
| enh17-F | 5′-AGGTTGAATATTTCCTC AAAGGCT-3′ |  |
| enh17-R | 5′-GGGGCACGAAGAATTCCACT-3′ |  |

**Supplemental** **Table S2.** **RNA-seq data sources and sample information.**

| **Data type** | **Data source** | **Sample** | **Tissue/cell line** |  |
| --- | --- | --- | --- | --- |
| **RNA-seq** | TCGA-SKCM | Primary and metastatic melanoma tumors | Tissue | Fig.S1A |
|  | GSE153592 | Invasive and non-invasive melanoma cell lines | cell line | Fig.S1B |
|  | GSE200217 | cell lines derived from patients with MBM or ECM | cell line | Fig.S1C |
|  | GSE35704 | Metastasis (A2058) and normal (HEMn) cell lines | cell line | Fig.S1D |
| **scRNA-seq** | GSE72056 | Primary and metastatic melanoma tumors | Tissue | Fig.S4 |
|  | GSE115978 | Primary and metastatic melanoma tumors | Tissue | Fig.S4 |
